# Vertically-Ordered Mesoporous Silica Films Grown on Boron Nitride-Graphene Composite Modified Electrodes for Rapid and Sensitive Detection of Carbendazim in Real Samples

**DOI:** 10.3389/fchem.2022.939510

**Published:** 2022-07-12

**Authors:** Yanqi Zou, Xiaoyu Zhou, Liuhong Xie, Hongliang Tang, Fei Yan

**Affiliations:** ^1^ Key Laboratory of Surface & Interface Science of Polymer Materials of Zhejiang Province, Department of Chemistry, Zhejiang Sci-Tech University, Hangzhou, China; ^2^ The First Clinical Faculty of Guangxi University of Chinese Medicine, Nanning, China; ^3^ Affiliated Fangchenggang Hospital, Guangxi University of Chinese Medicine, Fangchenggang, China

**Keywords:** vertically-ordered mesoporous silica films, boron nitride, graphene, carbendazim, electrochemical sensors, anti-fouling detection

## Abstract

Carbendazim (CBZ), a kind of widely used pesticide, is harmful to human health and environmental ecology. Therefore, it is of great importance to detect CBZ in real samples. Herein we report the stable growth of vertically-ordered mesoporous silica films (VMSF) on the glassy carbon electrode (GCE) using boron nitride-reduced graphene oxide (BN-rGO) nanocomposite as an adhesive and electroactive layer. Oxygen-containing groups of rGO and 2D planar structure of BN-rGO hybrid favor the stable growth of VMSF via the electrochemically assisted self-assembly (EASA) method. Combining the good electrocatalytic activity of BN-rGO and the enrichment effect of VMSF, the proposed VMSF/BN-rGO/GCE can detect CBZ with high sensitivity (3.70 μA/μM), a wide linear range (5 nM–7 μM) and a low limit of detection (2 nM). Furthermore, due to the inherent anti-fouling and anti-interference capacity of VMSF, direct and rapid electrochemical analyses of CBZ in pond water and grape juice samples are also achieved without the use of complicated sample treatment processes.

## Introduction

As a low-cost pesticide with broad spectrum activity, carbendazim (CBZ) plays a key role in controlling pests and diseases or weeds in agricultural production (Wei et al., 2018). Due to the stable characteristic of the benzimidazole ring, CBZ is stable and difficult to degrade. With the extensive and uncontrolled use of CBZ, the accumulation of CBZ residues can be found in the environment (e.g., soil and water), which may result in the long-term adverse effects on the ecological safety and water ecosystem (Ghorbani et al., 2021; Baigorria and Fraceto, 2022). Moreover, CBZ will cause serious health effects through the respiratory system and direct contacts, such as skin inflammation, eye irritation, disruption of the endocrine system, and hormonal disorder (Zhu et al., 2019; Farooq et al., 2020; Rai and Mercurio, 2020). Therefore, highly sensitive and accurate detection of CBZ in the environment is of significance for human health and environmental protection.

At present, sorts of methods for detecting CBZ have been developed, for example, mass spectrometry (Grujic et al., 2005), UV-vis spectroscopy (Pourreza et al., 2015), surface-enhanced Raman scattering (SERS) (Furini et al., 2015), gas chromatography (Lesueur et al., 2008), and high-performance liquid chromatography (Yamaguchi et al., 2011; Subhani et al., 2013). However, these techniques inevitably need expensive equipment, complex sample pretreatment steps, and a long analysis cycle (Yang et al., 2018). By contrast, the electrochemical method has the advantages of high sensitivity, low cost, rapidity, and convenient operation (Govindasamy et al., 2017a; Govindasamy et al., 2017b; Akilarasan et al., 2018; Keerthi et al., 2018), which has been applied for CBZ determination (Mekeuo et al., 2021). Due to the electrode fouling caused by the undesirable adsorption of biological macromolecules or microorganisms, complicated pretreatments of real samples are often required for electrochemical sensors and inevitably produce damage to the analytes. Therefore, designing anti-fouling and anti-interference electrode interfaces for direct electrochemical analysis of complex real samples is greatly important (Zhou et al., 2020).

Porous materials have aroused growing attention in the construction of rapid and portable sensors (Cui et al., 2020; Cui et al., 2021; Duan et al., 2021; Liu et al., 2022; Wei et al., 2022). Especially, vertically-ordered mesoporous silica films (VMSF) are a kind of porous material with perpendicularly ordered nanochannels and uniform pore size on a nanometer scale (Walcarius, 2021; Ma et al., 2022a). VMSF has advantages of high permeability, molecular selectivity, molecular sieving, insulation, and good mechanical and chemical stability, which has been widely employed as the anti-fouling coating for direct electrochemical analysis (Yan and Su, 2016; Huaxu Zhou et al., 2021). Arising from the high density of silanol groups on the channel walls and ultrasmall size of channels, molecular selectivity, and performance of VMSF could be modulated by modification of functional groups and confined growth of nanomaterials, broadening its practical applications (Lu et al., 2018; Yan et al., 2020a). For example, graphene quantum dots (GQD), a 0D graphene materials (Yan et al., 2019), are characterized by an atomically thin planar carbon structure with ultrasmall size (He et al., 2019; Mao et al., 2019), abundant active sites (Kaixin Li et al., 2018; Ge et al., 2019; Wan et al., 2021), tunable chemicophysical properties (Nan Li et al., 2018; Pang et al., 2018; Yan et al., 2018), and efficient heterogeneous electron transfer capacity (Huang et al., 2018; Tian et al., 2018; Gong et al., 2021a), could be confined into the nanochannels of VMSF to serve as the recognition, enrichment, and catalysis element, leading to the ultrasensitive electrochemical analysis of heavy metal ions and neurotransmitter in complex real samples (Lu et al., 2018). Currently, VMSF supported by indium tin oxide (ITO) electrode is rather stable and could be fabricated by using electrochemically-assisted self-assembly (EASA) and Stöber-solution approaches (Zhou et al., 2019; Walcarius, 2021). Premodification of molecular glues [e.g., organosilanes (Nasir et al., 2018; Gong et al., 2021b) or reduced graphene oxide (rGO) nanosheets (Yan et al., 2020b; Ma et al., 2022b)] and pretreatment process [e.g., electro-activation (Xuan et al., 2021; Wang et al., 2022) or plasma (Zhu et al., 2022)] can stably prepare VMSF on the other commercial conductive electrodes (e.g., metal electrode or carbonaceous electrode). As our group reported recently, the introduction of rGO as an adhesive and electroactive layer onto the electrode surface is capable of maintaining the well-oriented nanochannel structures of VMSF and greatly improving the sensitivity and selectivity of electrochemical sensors (Xi et al., 2019; Yan et al., 2021). Note that rGO hybrids with functional materials have been reported to further extend the scope of analytes (Kogularasu et al., 2017; Muthumariappan et al., 2017) and provide a versatile platform for VMSF-graphene-based electrochemical sensing platform (Lin Zhou et al., 2021; Zhou et al., 2022).

Thanks to the similar 2D planar structure to graphene, boron nitride (BN) has received more attention and it has several advantages of high specific surface area, good chemical and thermal stability, and excellent catalytic activity (Weng et al., 2016; Wang et al., 2021). Although the bulk BN is electrically insulating, the hybrid of graphene and BN could exhibit excellent electrochemical properties due to the smaller band gap (Qun Li et al., 2018). In this work, we report that VMSF could be stably grown onto the GCE by using a hybrid of BN and reduced graphene oxide (BN-rGO) as the adhesive and electroactive layer. BN-rGO modified GCE provides oxygen-containing groups, hydrophobic π-conjugated structure, and relatively planar substrate, favoring the stable growth of VMSF. And the obtained VMSF/BN-rGO/GCE displays good electrochemical performance to CBZ with high sensitivity and a low detection limit, owing to the excellent electrochemical activity of BN-rGO and enrichment effect of VMSF through strong hydrogen bonding. Furthermore, due to the excellent anti-fouling and anti-interference ability of VMSF, direct electrochemical analysis of CBZ in pond water and grape juice samples was achieved with good stability.

## Materials and Methods

### Chemical and Materials

All chemicals and reagents of analytical grade were used as received without further purification. And ultrapure water (18.2 MΩ cm) was used to prepare all aqueous solutions throughout this work. GO aqueous solution (1 mg/ml) was supplied from Hangzhou Gaoxi Tech. Boron nitride (99.9%), carbendazim (CBZ, 97%), Sodium phosphate monobasic dihydrate (NaH_2_PO_4_.2H_2_O, 99%), cetyltrimethylammonium bromide (CTAB), tetraethoxysilane (TEOS, 98%), potassium hydrogen phthalate (KHP, 99.8%), potassium ferricyanide (K_3_ [Fe(CN)_6_], 99.5%), humic acid (HA, 90%), starch soluble (Starch, 99%), hematin porcine (Heme, 95%), albumin from bovine serum (BSA, 96%), lauryl sodium sulfate (SDS, 98.5%) were gotten from Aladdin. Sodium phosphate dibasic dodecahydrate (Na_2_HPO_4_.12H_2_O, 99%) was obtained from Macklin. Lignin alkali was received from Solarbio. Sodium nitrate (NaNO_3_) was purchased from Wuxi Zhangwang Chemical Reagent. Hexaammineruthenium (III) chloride (Ru(NH_3_)_6_Cl_3_, 98%) was purchased from Sigma. Potassium chloride (KCl, 99.5%), calcium chloride (CaCl_2_, 95%), natrium bicarbonate (NaHCO_3_, 95%), sodium chloride (NaCl, 99.5%), magnesium chloride (MgCl_2_, 95%), potassium dihydrogen phosphate (KH_2_PO_4_, 99%) were obtained from Hangzhou Gaojing Fine Chemical Reagent. Pond water was obtained from the campus of Zhejiang Sci-Tech University. Grape juice was bought from supermarkets.

### Measurements and Instrumentations

X-Photoelectron spectroscopy (XPS) data was obtained from PHI5300 electron spectrometer (PE Ltd., United States) at 250 W, 14 kV, MgK α radiation. A transmission electron microscopy (TEM) image was collected from an HT7700 microscope (Hitachi, Japan) at an acceleration voltage of 100 kV. Cyclic voltammetry (CV) and differential pulse voltammetry (DPV) tests were performed on an Autolab (PGSTAT302N) electrochemical workstation (Metrohm, Switzerland) at room temperature. A routine three-electrode system was used, with Ag/AgCl (saturated with KCl) as the reference electrode, platinum electrode as the counter electrode, bare GCE, or modified GCE as the working electrode. The DPV arguments were as follows: step potential, 0.005 V; pulse time, 0.05 s; pulse amplitude, 0.05 V; interval time, 0.2 s.

### Synthesis of BN-rGO Dispersion

Boron nitride nanosheets (BN) were prepared according to the method previously reported with slight modification (Du et al., 2013). Briefly, 0.1 g of original boron nitride powder was dispersed into 5 ml H_2_SO_4_ (98%, w/w) and stirred for 30 min. Then, 0.1 g KMnO_4_ was added to the aforementioned solution at 0°C and stirred for another 12 h. After the addition of 0.5 ml of H_2_O_2_ (30%, w/w), the resulting suspension was centrifuged at 3,000 rpm for 5 min to remove the supernatant. After being washed with water and baked at 40°C for 24 h, BN was obtained.

0.0002 g BN was dispersed in 20 ml GO (0.1 mg/ml) solution and ultrasonicated for 0.5 h. Then, 60 μL ammonia water and 6 μL hydrazine hydrate (50%, w/w) were added to the aforementioned dispersion, followed by incubation in an aqueous bath at 60°C for 3.5 h. The obtained mixture was centrifuged at 3,000 rpm for 5 min, and the supernatant was taken to obtain the BN-rGO dispersion.

### Preparation of the VMSF/BN-rGO/GCE

**SCHEME 1:**
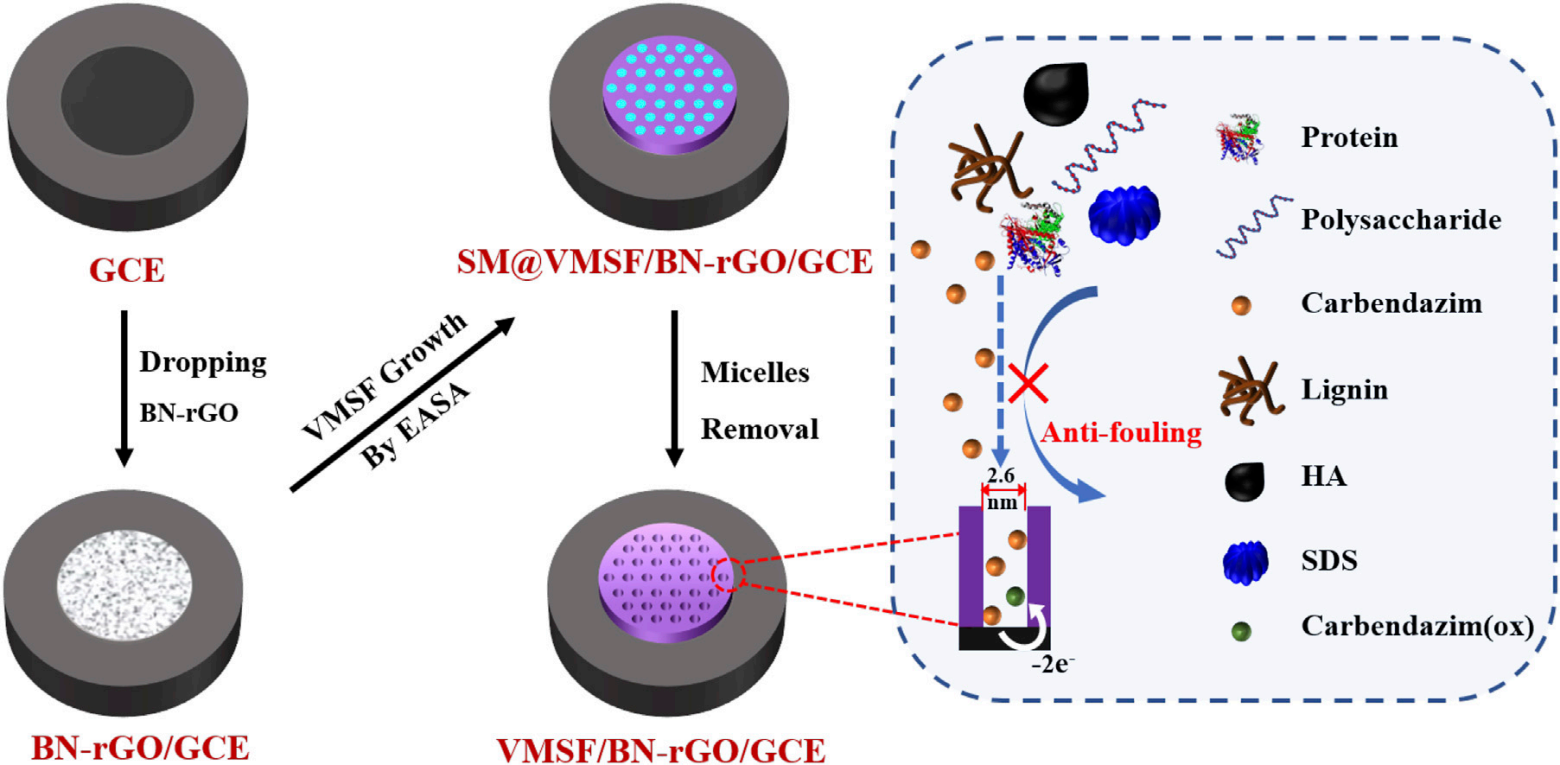
Illustration of the preparation of VMSF/BN-rGO/GCE electrode.

Prior to the electrode modification, GCE (3 mm diameter) was polished with 0.3 and 0.05 μm alumina powder, and then ultrasonically cleaned with absolute alcohol and distilled water. An illustration of the preparation of the VMSF/BN-rGO/GCE electrode is displayed in Scheme 1. As seen, 5 μl BN-rGO dispersion was primarily dropped onto a freshly cleaned GCE, and dried at 60°C. The resulting electrode was named BN-rGO/GCE. Then the VMSF was prepared on the BN-rGO/GCE by using the electrochemically assisted self-assembly (EASA) as previously reported (Xi et al., 2019). Briefly, a constant potential (–2.2 V) was applied to the BN-rGO/GCE for 5 s. After being aged at 80°C for 10 h, VMSF with the surfactant micelles (SM) inside the nanochannels was grown on the BN-rGO/GCE, termed as SM@VMSF/BN-rGO/GCE. Removal of SM could be performed by immersing the SM@VMSF/BN-rGO/GCE into the 0.1 M HCl-ethanol solution under moderate stirring for 5 min, to obtain VMSF/BN-rGO/GCE.

## Results and Discussion

### Characterization of VMSF/BN-rGO/GCE

**FIGURE 1:**
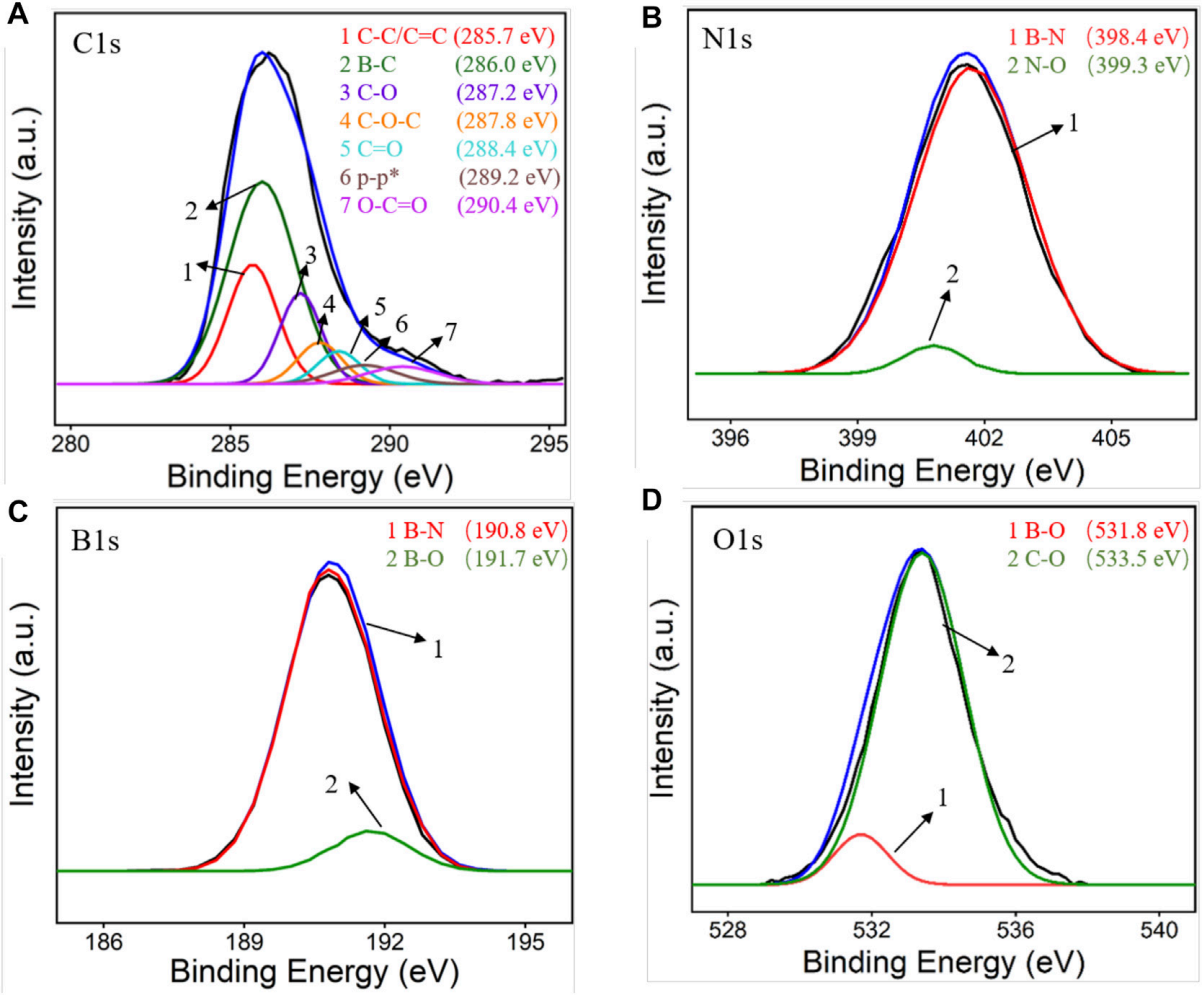
High resolution XPS spectra of BN-rGO: **(A)** C1s, **(B)** N1s, **(C)** B1s, and **(D)** O1s.

**FIGURE 2:**
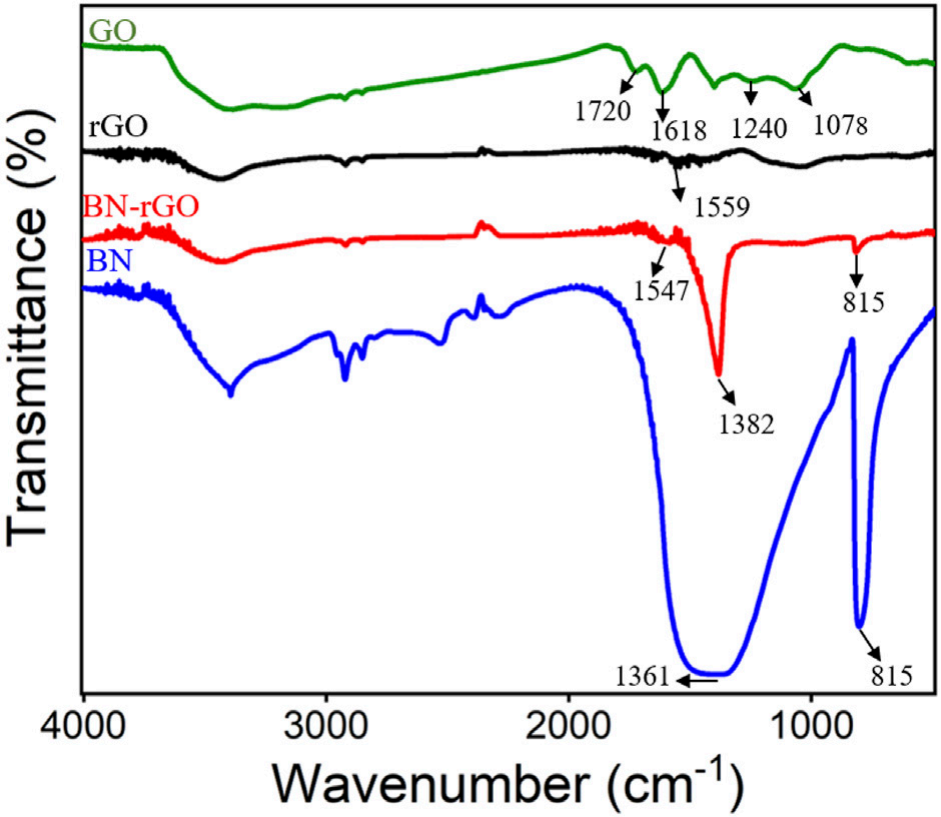
FT-IR spectra of GO, rGO, BN, and BN-rGO.

XPS was first employed to characterize the BN-rGO composite and the results were shown in Figure 1. As revealed in Figure 1A, there exist seven characteristic carbon 1 s XPS peaks located at 285.7, 286.0, 287.2, 287.8, 288.4, 289.2, and 290.4 eV, which are assigned to C-C/C=C, B-C, C-O, C-O-C, C=O, π-π* bond and O-C=O bond, respectively. Two obvious nitrogen 1 s XPS peaks were observed at 398.4 and 399.3 eV (Figure 1B), corresponding to B-N and N-O bonds, respectively. The B-N and B-O bonds of boron 1s produce two XPS peaks at 190.8 and 191.7 eV (Figure 1C). And two oxygen 1s XPS peaks corresponding to the B-O and C-O bonds are displayed at 531.8 and 533.5 eV. Figure 2 depicts the FT-IR spectra of the BN-rGO composite. It could be found that GO has four characteristic peaks at ∼1,078 cm^−1^ (C-O), ∼1,240 cm^−1^ (C-O-C), and ∼1,618 cm^−1^ (C=C), and ∼1720 cm^−1^ (C=O). After the chemical reduction of GO to rGO, intensities of oxygen-containing groups remarkably decrease and an absorption peak at ∼1,559 cm^−1^ (C=O) is observed. In comparison with BN and rGO, BN-rGO nanocomposite possesses characteristic absorption bands of BN at 815 cm^−1^ (B-N), ∼1,382 cm^−1^ (B-N-B), and that of rGO at 1,547 cm^−1^ (C-O), showing the successful preparation of BN-rGO nanocomposite.

**FIGURE 3:**
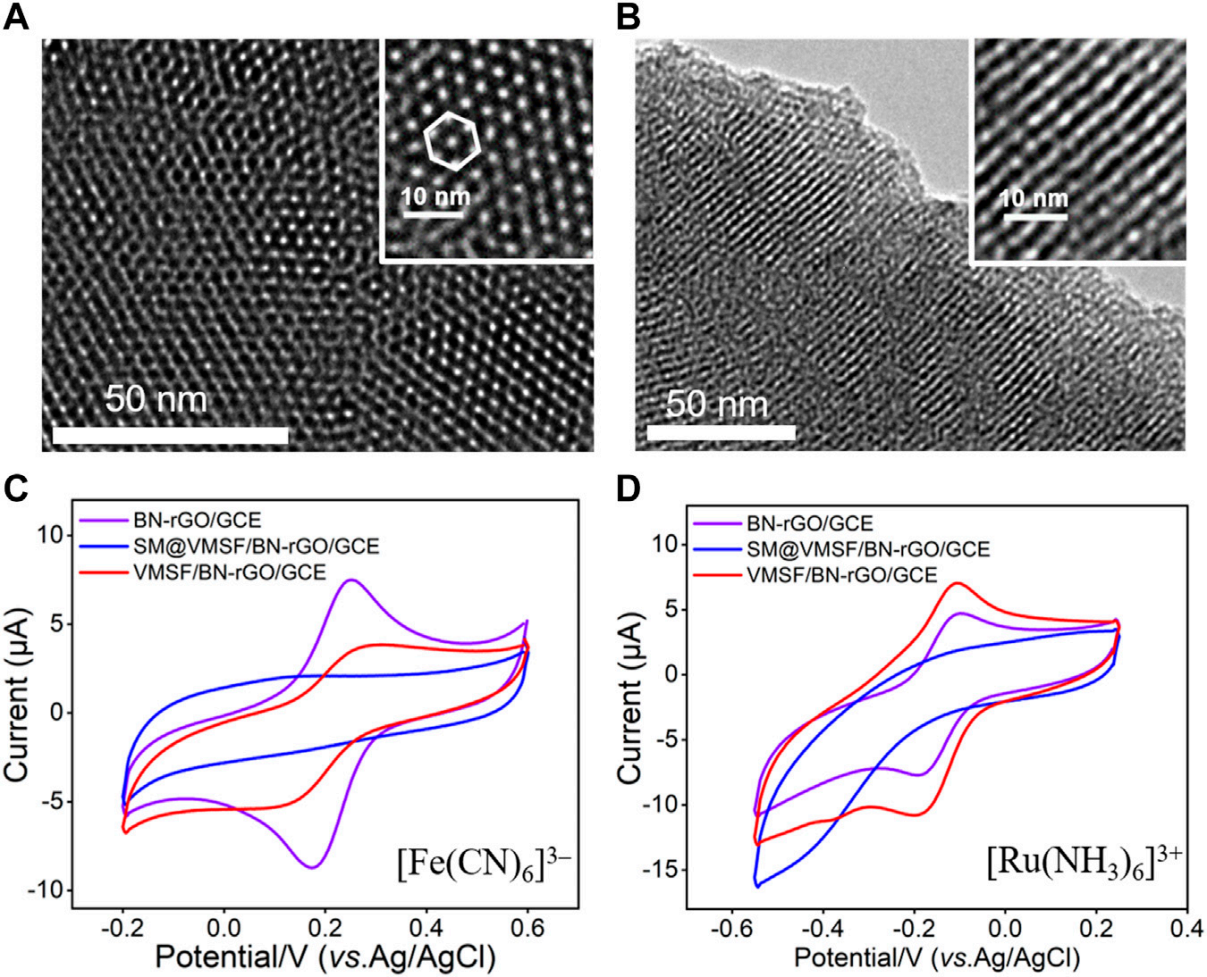
Top-view **(A)** and cross-sectional view **(B)** TEM images of VMSF. The insets are corresponding magnified images. CV curves obtained from the BN-rGO/GCE, SM@VMSF/BN-rGO/GCE and VMSF/BN-rGO/GCE electrodes in 0.05 M KHP containing 0.5 mM [Fe(CN)_6_]^3–^ **(C)** and [Ru(NH_3_)_6_]^3+^ **(D)**. The scan rate was 50 mV/s.

VMSF was grown onto the BN-rGO/GCE by using the EASA method (Xi et al., 2019) and its morphology was characterized by TEM. It can be seen from the top-view TEM image that VMSF has hexagonal and regular nanopores with a uniform pore diameter of 2.6 nm (Figure 3A). And cross-sectional TEM image reveals that the nanochannels of VMSF are perpendicularly oriented and parallel to each other (Figure 3B). The integrity and permeability of VMSF were investigated by cyclic voltammetry (CV) using two kinds of conventional charged electrochemical probes, namely positively charged Ru(NH_3_)_6_^3+^ and negatively charged Fe(CN)_6_^3–^. As shown in Figures 3C, D, no obvious redox signals for both Ru(NH_3_)_6_^3+^ and Fe(CN)_6_^3–^ are observed at the SM@VMSF/BN-rGO/GCE, which is due to the impermeable SM inside the nanochannels of VMSF and further indicates the obtained VMSF onto the BN-rGO/GCE is intact. After the extraction of SM from the nanochannels, electrochemical signals of two charged probes are recovered to a certain extent at the VMSF/BN-rGO/GCE. And VMSF/BN-rGO/GCE displays apparent charge permselectivity, namely attracting Ru(NH_3_)_6_^3+^ and repelling Fe(CN)_6_^3–^, compared to the BN-rGO/GCE. This is because silanol groups onto the inner walls of VMSF are deprotonated to produce a negative charge under the experimental condition. Note that the current magnitude of Ru(NH_3_)_6_^3+^ at the VMSF/BN-rGO/GCE is comparable to that of the BN-rGO/GCE, suggesting the high permeability of VMSF.

### Electrochemical Behavior of VMSF/BN-rGO/GCE

**FIGURE 4:**
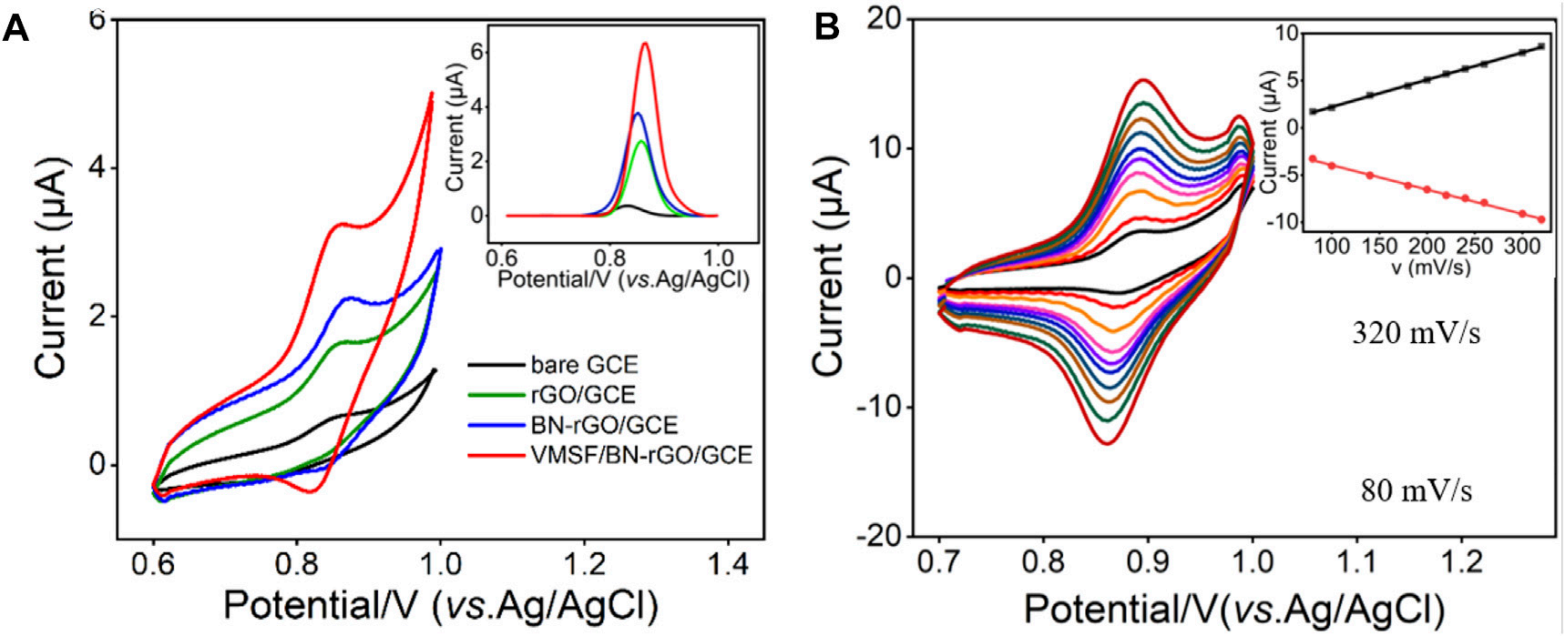
**(A)** CV curves obtained from the bare GCE, rGO/GCE, BN-rGO/GCE, and VMSF/BN-rGO/GCE electrodes in 0.1 M PBS (pH 6.0) containing 1 μM CBZ. Inset is the corresponding DPV curves. The scan rate was 50 mV/s. **(B)** CV curves of 1 μM CBZ in 0.1 M PBS (pH 6.0) at the VMSF/BN-rGO/GCE at different scan rates (80–320 mV s^−1^). The inset is the relationship between the oxidation peak currents and scan rates.

**SCHEME 2:**
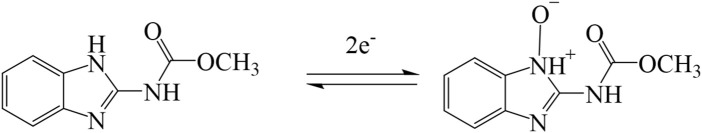
Electrochemical reaction mechanism of CBZ.

In order to testify the detection performance, CV and DPV responses of 1 μM CBZ at the bare GCE, rGO/GCE, BN-rGO/GCE, and VMSF/BN-rGO/GCE were compared in Figure 4A. As shown, CBZ can produce weak redox peaks at the bare GCE electrode, corresponding to the redox reaction of CBZ (Scheme 2) (Cui et al., 2017). After modification of rGO on the GCE electrode, remarkably increased current signals are observed at the rGO/GCE, suggesting the good electrocatalytic activity of rGO. The introduction of BN into the rGO nanosheets could further enhance the current signals, indicating the higher electrocatalytic activity of the BN-rGO composite. Due to the enrichment effect of hydrogen bonds between the silanol groups of VMSF and secondary amine groups of CBZ, peak currents obtained at the VMSF/BN-rGO/GCE further increase. And the magnitude of oxidation peak current at the VMSF/BN-rGO/GCE is about 2-3-fold higher than that obtained at the rGO/GCE or BN-rGO/GCE and 30-fold higher than that obtained at the bare GCE (inset of Figure 4A). Figure 4B shows the CV curves of 1 μM CBZ at the VMSF/BN-rGO/GCE at various scan rates. As demonstrated, both oxidation and reduction peak currents have a good linear relationship with scan rate in the range of 80–320 mV s^−1^, indicating the adsorption-controlled electrochemical process.

### Optimization of Experimental Conditions

To achieve the optimal detection performance, the influences of pH value of supporting electrolyte and preconcentration time on the electrochemical detection of CBZ were studied. Supplementary Figure. S1A shows the DPV responses of the VMSF/BN-rGO/GCE to 1 μM CBZ in 0.1 M PBS at different pH values. With the pH increasing from 4.0 to 8.0, the oxidation peak shifts negatively and exists a favorable linear relationship with the pH (inset of Supplementary Figure S1A) with the slope of −62 mV/pH. This suggests that the electron transfer is accompanied by an equal number of protons in the redox reaction of CBZ at the VMSF/BN-rGO/GCE according to the Nernst equation (Ilager et al., 2021). Moreover, the maximal oxidation peak current is obtained at the pH of 6.0, which is used for subsequent detection of CBZ. Since mechanical stirring could accelerate the diffusion of CBZ to the underlying electrode surface along the nanochannels of VMSF, the influence of stirring time on detection performance was investigated. As displayed in Supplementary Figure S2, the oxidation peak current of CBZ at the VMSF/BN-rGO/GCE increases with the increasing stirring time and reaches a plateau at 4 min. Therefore, 4 min was employed as the best preconcentration time for the following study.

### Electrochemical Determination of CBZ in Buffer Solution

**FIGURE 5:**
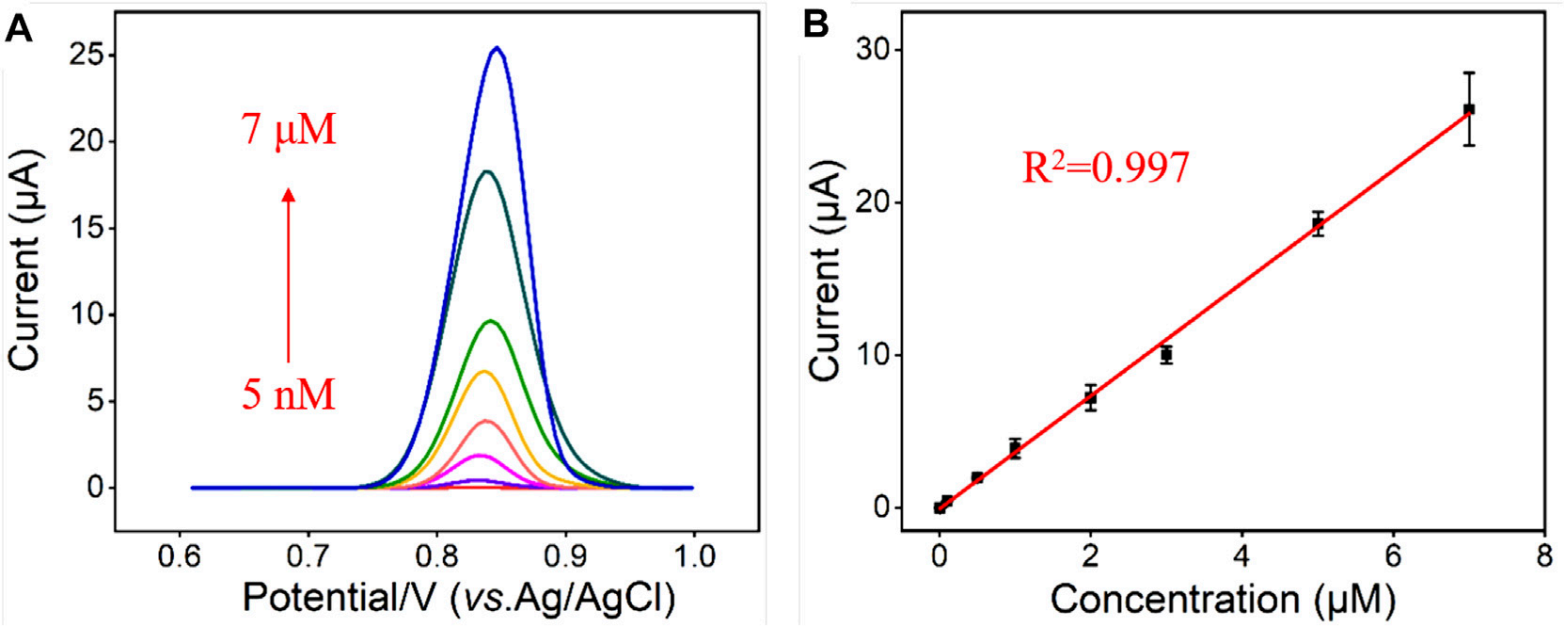
**(A)** DPV curves obtained from the VMSF/BN-rGO/GCE in response to different concentrations of CBZ (0.005, 0.01, 0.1, 0.5, 1, 2, 3, 5 and 7 μM). The supporting electrolyte is 0.1 M PBS (pH 6.0); Inset is amplified DPV curves. **(B)** Calibration plot for CBZ. The error bars represent the standard deviation (SD) of three measurements.

Under optimal experimental conditions, VMSF/BN-rGO/GCE was utilized to detect CBZ with different concentrations and the results were shown in Figure 5. As can be seen, as the CBZ concentrations increase, the measured oxidation peak current signals increase gradually in the range from 5 nM to 7 μM. There is a good linear relationship between oxidation peak currents and CBZ concentration. And the obtained linear fitting equation was *I* (μA) = 3.70 C (μM)—0.0512 (*R* ^2^ = 0.997), with a limit of detection (LOD) of 2 nM. Table 1 compares the analytical performances between the proposed VMSF/BN-rGO/GCE and other reported sensors for CBZ detection. As presented, VMSF/BN-rGO/GCE has a relatively wide linear range and a lower LOD.

**TABLE 1:** Comparison of the analytical performances of various analytical methods for the determination of CBZ.

Materials	Method	Range (μM)	LOD (μM)	Ref
N, P-CQDs, and Au NPs	FL	0.005–1.57	0.002	Yang et al. (2018)
N-CQDs/AuNCs	FL	1–100	0.83	Yang et al. (2020)
	SERS	150–1,000	37.85	
SAX/PSA	HPLC-UV	0.26–1.57	0.015	Phansawan et al. (2015)
NPG/GCE	Electrochemistry	10–70	0.24	Gao et al. (2019)
CMC-MWCNTs/GCE	Electrochemistry	0.03–10	0.015	Zhou et al. (2018)
CPE/FS@Ag	Electrochemistry	0.05–10	0.00094	Ozcan et al. (2021)
NP-Cu/rGO/GCE	Electrochemistry	0.5–30	0.09	Tian et al. (2019)
VMSF/BN-rGO/GCE	Electrochemistry	0.005–7	0.002	This work

N, P-CQDs: N, P-doped carbon quantum dots; Au NPs: FL, fluorescence; gold nanoparticles; N-CQDs: nitrogen-doped carbon quantum dots; AuNCs: gold nanocluster; SAX/PSA: strong anion exchange/primary secondary amine; NPG: nanoporous gold; CMC: carboxymethyl cellulose; MWCNTs: multi-walled carbon nanotube; CPE: carbon paste electrode; FS: silver nanoparticles on fumed silica; NP-Cu: nanoporous copper.

### Anti-interference and Anti-fouling Performance

**FIGURE 6:**
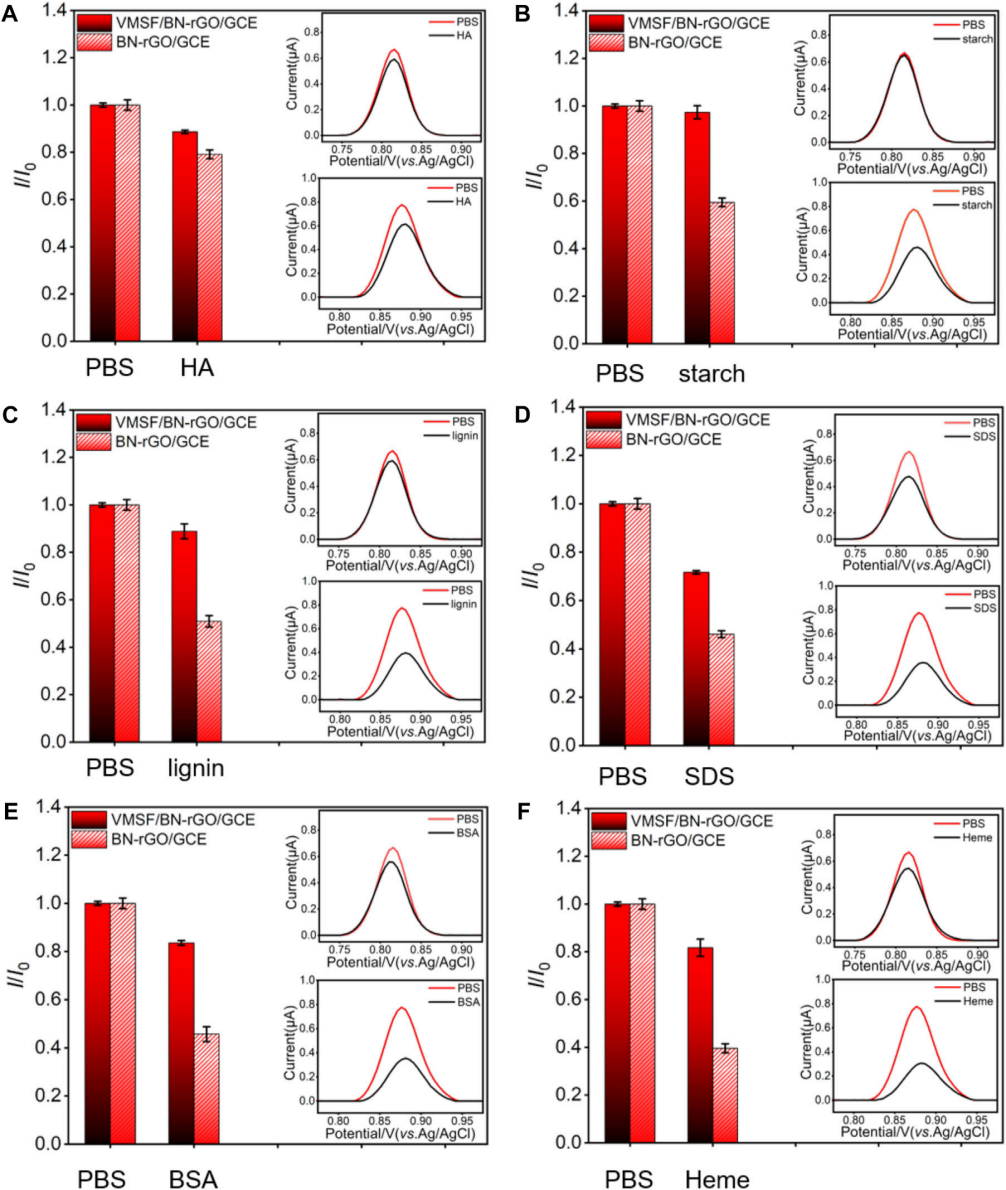
Current ratio (*I*/*I*_0_) obtained from VMSF/BN-rGO/GCE and BN-rGO/GCE for the detection of 1 μM CBZ in 0.1 M PBS containing 50 μg/ml HA **(A)**, starch **(B)**, lignin **(C)**, SDS **(D)**, BSA **(E)**, or Heme **(F)**. Insets are corresponding DPV curves of VMSF/BN-rGO/GCE (red line) and BN-rGO/GCE (black line) in the absence (top) or presence (bottom) of fouling species. The error bars represent the SD of three measurements.

Due to the intrinsic anti-interference and anti-fouling capacities, we investigated the performance of the VMSF/BN-rGO/GCE by comparing the oxidation peak currents of CBZ in the absence and presence of various ions (CO_3_^2-^, PO_4_^3-^, Mg^2+^, K^+^_,_ and Na^+^) and biologically related species (HA, starch, lignin, SDS, BSA, and heme). The results shown in Supplementary Figure S3 and Figure 6 suggest that the presence of interferents has no obvious influence on the CBZ detection. Moreover, the anti-fouling performance of the VMSF/BN-rGO/GCE and BN-rGO/GCE was compared in Figure 6. As seen, BN-rGO/GCE has a much reduced oxidation peak current after the addition of biologically related species into the buffer solution. By contrast, VMSF/BN-rGO/GCE remains comparable signals for these six interfering species, indicating the great potential of VMSF/BN-rGO/GCE in complex samples. However, arising from the hydrolysis of VMSF, the proposed sensor could not be used in strong alkaline solutions for a long time.

### Electrochemical Determination of CBZ in Real Samples

We selected pond water and grape juice as actual samples to investigate the feasibility of the sensor in practical application. The pond water (20-fold diluted) and grape juice (50-fold diluted) samples were only diluted by 0.1 M PBS (pH = 6.0) prior to determination. Then a series of CBZ solutions with known concentrations were added to the aforementioned diluted pond water and grape juice samples. By comparing the detected concentrations detected by VMSF/BN-rGO/GCE with the known concentrations, good recoveries, and low RSD values are observed at the VMSF/BN-rGO/GCE, proving that the proposed sensor can quantitatively detect CBZ in real samples.

## Conclusion

In summary, we have reported a simple electrochemical method for highly sensitive detection of CBZ using the VMSF/BN-rGO/GCE sensor. A layered nanocomposite consisting of BN and rGO could act as a conductive and stabilized layer for the stable growth of VMSF by using the EASA method. Arising from the excellent electrocatalytic performance of BN-rGO nanocomposite and the good anti-fouling capacity, the proposed VMSF/BN-rGO/GCE sensor can realize the direct and highly sensitive detection of CBZ in complex sample of pond water and grape juice samples. Integration with flexible electrodes and wireless devices will make the present sensor more useful in environmental monitoring and food quality control (Table 2).

**TABLE 2:** Recovery of CBZ in diluted pond water and grape juice.

Sample	Added (μM)	Found (μM)	RSD (%)	Recovery (%)
Pond water	0.300	0.313	3.3	104
	1.00	0.976	0.7	97.6
	2.00	2.04	0.2	102
Grape juice	0.300	0.303	4.0	101
	1.00	0.963	2.0	96.3
	2.00	2.02	2.1	101

## Data Availability

The original contributions presented in the study are included in the article/Supplementary Material, further inquiries can be directed to the corresponding authors.
