# Supplementary material for: Vertically-Ordered Mesoporous Silica Films Grown on Boron Nitride-Graphene Composite Modified Electrodes for Rapid and Sensitive Detection of Carbendazim in Real Samples
Source: Front Chem. 2022 Jul 12;10:939510. doi: 10.3389/fchem.2022.939510 (PMC9314778; doi:10.3389/fchem.2022.939510)
Supplement: Supplementary file 1 [file DataSheet1.docx]

Vertically-Ordered Mesoporous Silica Films Grown on Boron Nitride-Graphene Composite Modified Electrodes for Rapid and Sensitive Detection of Carbendazim in Real Samples

**Yanqi Zou^a^, Xiaoyu Zhou^a^, Liuhong Xie^b^, Hongliang Tang^c^* and Fei Yan^a^***

^a^ Key Laboratory of Surface & Interface Science of Polymer Materials of Zhejiang Province, Department of Chemistry, Zhejiang Sci-Tech University, Hangzhou, China.

^b^ Guangxi University of Chinese Medicine, Nanning, China.

^c^ Affiliated Fangchenggang Hospital, Guangxi University of Chinese Medicine, Fangchenggang, China.

*** Correspondence:**

Corresponding authors: tanghongliang@gxtcmu.edu.cn; feifei19881203@126.com

Fei Yan: 0000-0002-2822-698X

**Table of Contents**

S1 Optimization of experimental conditions

S1.1 pH of supporting electrolyte

S1.2 Preconcentration time

S2 Anti-interference ability of VMSF/BN-rGO/GCE

**S1 Optimization of experimental conditions**

**S1.1 pH of supporting electrolyte**

**
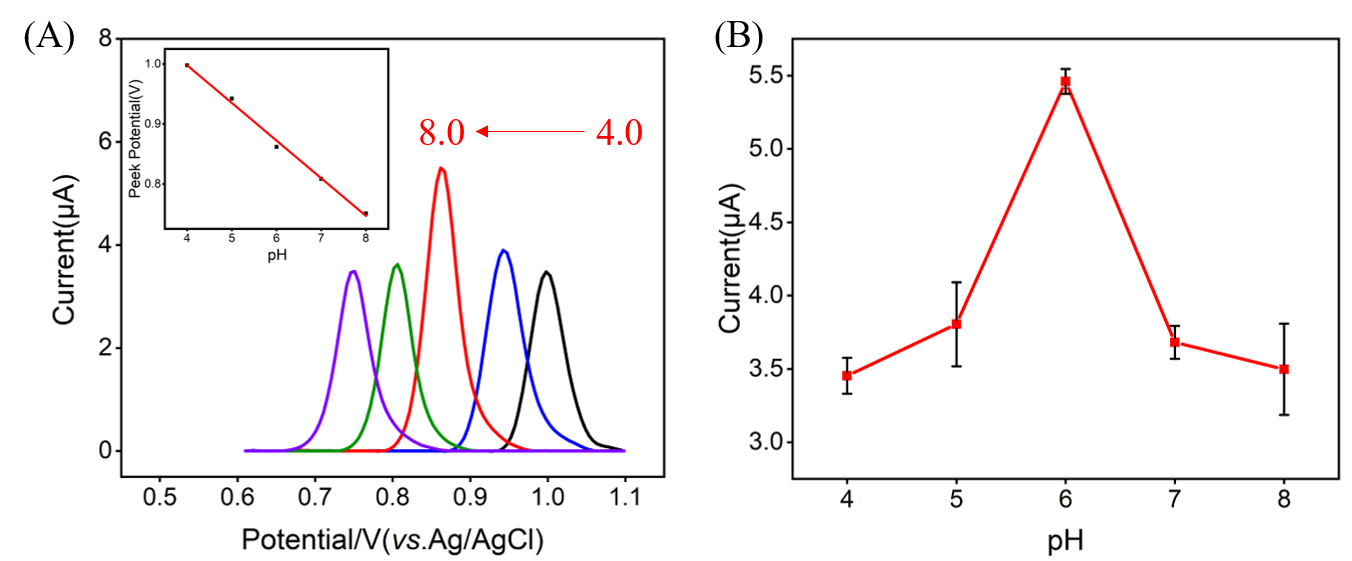
**

**Fig. S1** (A) DPV of 1 μM CBZ in 0.1 M PBS at the VMSF/BN-rGO/GCE with different pH values (4.0–8.0). Insert shows the relationship between the oxidation peak potential and pH. (B) Effect of the pH value of PBS on the detection of 1 μM CBZ at the VMSF/BN-rGO/GCE. The error bars represent the SD of three measurements.

**S1.2 Preconcentration time**

**
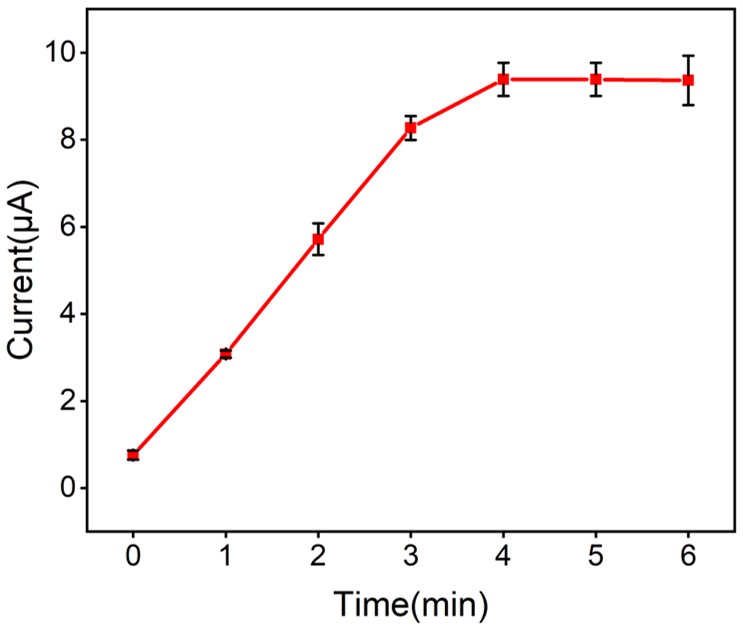
**

**Fig. S2** Effect of mechanical stirring time on the detection of 1 μM CBZ in 0.1 M PBS (pH 6.0) at the VMSF/BN-rGO/GCE. The error bars represent the SD of three measurements.

**S2 Anti-interference ability of VMSF/BN-rGO/GCE**

**
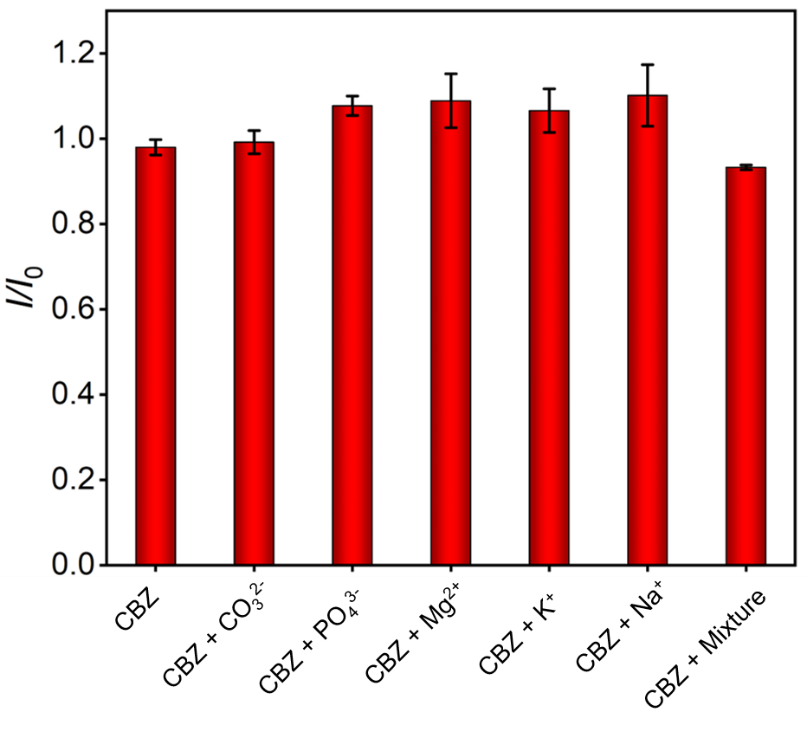
**

**Fig. S3** Oxidation peak current ratio (*I*/*I*_0_) obtained from the VMSF/BN-rGO/GCE in 0.1 M PBS (pH 6.0) containing 1 μM CBZ in the absence (*I*_0_) or presence (*I*) of 5 μM various interfering ions.
